# Competition for nutrients and its role in controlling immune responses

**DOI:** 10.1038/s41467-019-10015-4

**Published:** 2019-05-09

**Authors:** Nidhi Kedia-Mehta, David K. Finlay

**Affiliations:** 1 School of Biochemistry and Immunology, Trinity Biomedical Sciences Institute, Trinity College Dublin, 152-160 Pearse Street, Dublin, D02 R590 Ireland; 2 School of Pharmacy and Pharmaceutical Sciences, Trinity Biomedical Sciences Institute, Trinity College Dublin, 152-160 Pearse Street, Dublin, D02 R590 Ireland

**Keywords:** Immunology, Cell death and immune response, Signal transduction, Metabolism

## Abstract

Changes in cellular metabolism are associated with the activation of diverse immune subsets. These changes are fuelled by nutrients including glucose, amino acids and fatty acids, and are closely linked to immune cell fate and function. An emerging concept is that nutrients are not equally available to all immune cells, suggesting that the regulation of nutrient utility through competitive uptake and use is important for controlling immune responses. This review considers immune microenvironments where nutrients become limiting, the signalling alterations caused by insufficient nutrients, and the importance of nutrient availability in the regulation of immune responses.

## Abstract (web summary)

Immune cells adapt distinct metabolic strategies to accommodate specific functions associated with cell types or differentiation stages. Here in this review the authors discuss the nutrients, sensors, and mediators of such a metabolic adaption in nutrient-limiting immune microenvironments such as tumors or infections.

## Introduction

**Fig. 1:**
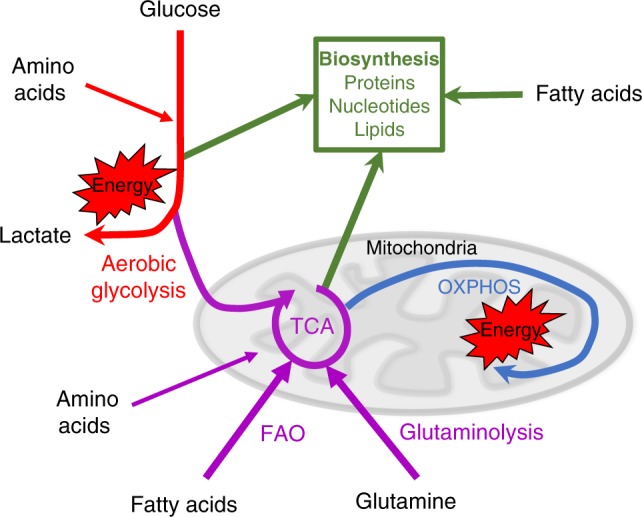
Metabolism configured to support energy homoeostasis and biosynthesis. Cellular metabolism can be configured to efficiently generate energy in the form of ATP. Glucose is metabolised by aerobic glycolysis (red) and via glycolysis coupled to the tricarboxylic acid (TCA) cycle (purple) to drive oxidative phosphorylation (OXPHOS) (blue) and the generation of energy in the form of ATP. Additional fuels, including fatty acids and the amino acid glutamine, can be used to support OXPHOS. Various other amino acids can also feed into both glycolysis and the TCA cycle. In addition to fuelling energy production, glucose and amino acids can be metabolised and used to support biosynthetic processes (green). Intermediates of glycolysis and the TCA cycle can be diverted into metabolic pathways to generate biosynthetic precursors important for the synthesis of lipids, nucleotides and proteins. Fatty acids can also be directly used for biosynthesis

Immune responses involve rapid and extensive changes in the activities of immune cells with concomitant alterations in cellular metabolism. Immune cells have various demands for nutrients, including glucose, glutamine and fatty acids, which are metabolised to generate ATP for energy expenditure. Meanwhile, these nutrients are also used to generate cellular building blocks for the biosynthesis of cellular components, including protein, nucleic acids and lipids (Fig. 1). Indeed, diverse metabolic configurations supported by a number of different nutrients have been described for immune subsets, which facilitate the specialised immune functions of individual cells^1^. To understand how distinct immune cells are affected by immune microenvironments where nutrient levels are limited, it is important to first appreciate the nutrient demands and metabolic configurations of different immune cells, as briefly outlined in the first section of this review [reviewed in detail elsewhere^1–3^]. We then discuss the circumstances in which nutrients might become limiting within different types of immune microenvironment, including the tumour microenvironment and sites of infection. Finally, we consider the consequences of nutrient deprivation on nutrient-sensitive signalling pathways and its impact for immune function.

## Nutrient demands and metabolic configurations

**Fig. 2:**
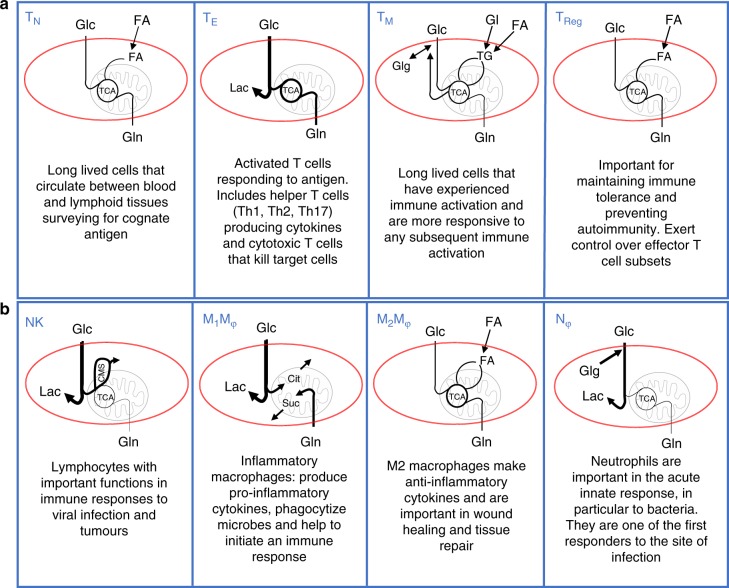
Illustrating the different metabolic configurations of immune cells. **a** T cells: Naive T cells (T_N_) have low metabolic rates fueled by glucose and glutamine. Effector T cell (T_E_) subsets tend to have elevated levels of both aerobic glycolysis (for metabolising glucose to lactate (Lac)) and OXPHOS (as fueled by glucose (Glc) and glutamine (Gln)). Memory T cells (T_M_) maintain intracellular fuel stores in the form of glycogen (Glg) and triacylglycerides (TG) fueled by glucose and fatty acid (FA) uptake, and primarily use OXPHOS rather than glycolysis. T_M_ have metabolic plasticity as they can engage multiple opposing metabolic pathways including gluconeogenesis/glycolysis, glycogenesis/glycogenolysis and FA synthesis/FA oxidation. TG stores are generated using imported glycerol (Gl). This metabolic configuration supports two key features of T_M_ cells; long term survival by providing dependable fuel sources within the cell (TG and Glg) and rapid metabolic responses to re-stimulation because the metabolic machinery is already present and in use. Regulatory T cells (T_Reg_) import FA for use in biosynthesis and to generate energy through FA oxidation. **b** Other immune cells: Natural killer (NK) cells primarily use glucose as a fuel, which supports aerobic glycolysis and drives OXPHOS through the citrate-malate shuttle (CMS) but not the TCA cycle. In M1 macrophages (M_1_M_φ_) the TCA cycle is broken, and glucose is metabolised to lactate and citrate (Cit) (used to make immunoregulatory molecules such as itaconate) while glutamine is metabolised to succinate (Suc) (used to generate mitochondrial ROS). By contrast, M2 macrophages (M_2_M_φ_) maintain an intact TCA and favour oxidative metabolism that is fuelled by the uptake of fatty acids, glutamine and glucose. Neutrophils primarily use glycolysis fuelled by glucose uptake and internal glycogen stores, and have very low OXPHOS

Cellular metabolism is a key factor in determining the fate and functions of immune cells. Studies have shown that disrupting metabolic signalling pathways can result in the loss of normal immune function or altered immune cell differentiation. Immune cells have diverse functions, so it is perhaps unsurprising that they have different nutrient and energy demands. As T cells differentiate into memory, regulatory and effector T cell subsets, the nutrients they use and the metabolic configurations they adopt adjust to match their respective metabolic demands. A good example is the different metabolic requirements of naive and effector T cell subsets (Fig. 2)^4^. Naive T cells have low metabolic rates and minimal biosynthetic requirements when compared to activated T cell subsets, which is due to their relative inactivity. Naive T cells take up small amounts of fuels such as glucose, glutamine and fatty acids, which they metabolise through oxidative phosphorylation (OXPHOS), primarily for the purpose of making energy (Figs. 1 and 2)^4^. Metabolic rates significantly increase in T cells following immune activation due to proliferative expansion and the induction of a range of effector functions including the production of large amounts of cytokines, a process that demands substantial amounts of energy and cellular biosynthesis. This leads to an increased demand for nutrients, including glucose and glutamine as well as amino acids such as serine and arginine, to fuel both bioenergetic and biosynthetic pathways (Figs. 1 and 2)^4–8^. Effector T cells have high rates of glucose and glutamine uptake, which are then metabolised by aerobic glycolysis in the cytoplasm and the tricarboxylic acid (TCA) cycle in the mitochondria^9–11^. Effector T cells also have increased uptake of other amino acids including, but not limited to, leucine, serine and tryptophan^6,7^. This metabolic configuration supports the combined cellular needs of effector T cells for energy and biosynthesis (Fig. 1). By contrast, memory T cells return to a quiescent state and have reduced biosynthetic demands, and revert to using oxidative metabolism for more efficient energy production. Memory T cells also generate intracellular fuel reserves in the form of glycogen and triacylglycerides that provide them with metabolic security and plasticity essential to support the longevity and rapid recall responses that are central to their functions^4,5^. Regulatory T cells, important in exerting control over effector T cells, do not have large biosynthetic demands and so predominantly engage in OXPHOS, which is fuelled by exogenous fatty acids imported and metabolised via a pathway called fatty acid oxidation to generate energy, (Fig. 1)^12^. It should be noted that when regulatory T cells do engage in cellular division, they switch on glycolytic metabolism to support the biosynthetic demands for growth and proliferation^13,14^. Disrupting cellular metabolism in T cells results in impaired T cell function and alters the differentiation of T cells towards effector, memory or regulatory subsets^3^.

B lymphocytes, or B cells, are important for humoral immunity and are the cells that produce antibodies. B cells also up-regulate glucose uptake and metabolic genes upon B cell antigen receptor activation to fuel both energy production and biosynthesis; in particular, de novo lipogenesis is required during the differentiation of plasma cells, which are responsible for making large amounts of high affinity antibody^15,16^. Glutamine can also be used by B cells and is particularly important for B cell survival in hypoxic conditions^17^. Other lymphocytes, such as activated natural killer (NK) cells are fuelled primarily by glucose but not by glutamine (Fig. 2). Here glucose simultaneously supports high rates of aerobic glycolysis and mitochondrial respiration, with the latter being achieved by engaging the citrate–malate shuttle, rather than the TCA cycle, to drive OXPHOS^18,19^. It is not yet clear why NK cells adopt this metabolic configuration but it seems likely that it is important to support epigenetic regulation as the citrate–malate shuttle also generates acetyl-CoA, the substrate for histone acetylation.

Myeloid cells have also been reported to adopt distinct metabolic phenotypes and have differing requirements for nutrients. Inflammatory M1 macrophages adopt a glycolytic metabolism and largely shut down OXPHOS; the TCA cycle is not operating in these cells, and the TCA cycle enzymes are repurposed into two linear metabolic pathways that produce important immune regulatory molecules, meaning that this metabolic configuration is essential for the proinflammatory functions of these cells (reviewed in ref. ^20^). In contrast, M2 macrophages, which are longer lived than M1 macrophages, are implicated in wound healing and tissue repair, and maintain an intact TCA cycle and favour oxidative metabolism fuelling OXPHOS with glutamine and fatty acids^2^. In this regard, interfering with macrophage metabolism can alter the differentiation of M1 versus M2 macrophages, such that the inhibition of glycolysis promotes the differentiation of M2 macrophages over M1 macrophages^3^. Lastly, the amino acid arginine is important for both M1 and M2 macrophages as a substrate for the enzymes inducible nitric oxide synthase (iNOS) and arginase, respectively.

Granulocytes such as neutrophils have very low levels of OXPHOS and rely primarily on glycolysis^3^. Interestingly, inflammatory neutrophils contain large glycogen deposits that are intracellular fuel stores that can be used to sustain this glycolytic metabolism in the absence of glucose^21^. This may provide neutrophils with an advantage in inflammatory microenvironments where extracellular glucose levels are limiting. Dendritic cells (DCs) have also been shown to contain glycogen stores that are important in fuelling the immediate glycolytic response following lipopolysaccharide stimulation^22^. Interestingly, glycolytic restriction can both inhibit and enhance DC functions depending on its timing during DC activation. Inhibition of glycolysis during initial activation results in impaired DC function, while inhibition of glycolysis 8 h after initial activation, a time point when DCs are likely to have reached the draining lymph node, enhances DC proinflammatory function and induction of T cell responses^22–25^.

Overall, it is clear that different immune cell subsets have distinct demands for nutrients, so they will be differentially affected by nutrient-restrictive microenvironments such as tumours or sites of infection. This argues that nutrient availability could play an important role in shaping immune responses.

## Availability of nutrients within immune microenvironments

Tumours have long been known to be highly glycolytic and to have a prodigious appetite for glucose, which is used to support unrestrained tumour growth and proliferation. This elevated glucose utilisation by tumour cells rapidly consumes the glucose delivered to the tumour from the blood resulting in low extracellular levels of glucose within solid tumours^26–28^. Some tumours also rely on glutamine to support their energy and biosynthetic demands^29,30^, and there is some evidence to suggest that glutamine levels may become limiting in some tumour microenvironments^31^. These features make the tumour microenvironment a metabolically restrictive setting for infiltrating immune cells, and this has been reported to have an effect on the efficacy of cancer immunotherapies; in the case of human melanoma, tumour cells with high glycolytic rates have been found to be resistant to adoptive T cell therapy showing poor T cell infiltration and cytotoxicity^32^.

Do similar mechanisms to those observed in the tumour microenvironment affect immune cells at other immunological sites? There is certainly evidence that competition for nutrients is also relevant at sites of pathogen infection. Many viruses reprogramme the cells that they infect for increased glycolysis^33–39^, and some have been reported to increase glutamine metabolism in the cells that they infect^40,41^. Increased glycolysis and/or glutaminolysis are advantageous for the virus, as they provide the biosynthetic material to construct new viral particles and complete the viral life cycle. Similarly, intracellular bacteria, such as *Mycobacterium tuberculosis*, also reprogramme the host cell for increased glucose metabolism and glycolysis^42–45^. Increased fuel consumption in these infected cells is likely to lead to decreased concentrations of extracellular glucose and glutamine in the local microenvironment. Additionally, many extracellular bacteria, such as *Staphylococcus aureus*, use glucose as their primary fuel source, and large numbers of bacteria can accumulate at a given site during infection^46^. Therefore, it is likely that glucose levels will become depleted at such sites of bacterial infection. Indeed, reduced glucose levels are reported in patients with bacterial meningitis^47^. Therefore, it is likely that at sites of infection, the increased utilisation of glucose and glutamine by bacteria or virally infected cells will affect responding immune cells through decreased nutrient availability in the local immune microenvironment.

Beyond the competitive utilisation of glucose and glutamine, the levels of other nutrients can be manipulated within immune microenvironments. Tumours can deplete amino acids, such as arginine and tryptophan, from the tumour microenvironment by expressing catabolic enzymes or by recruiting cells that express such enzymes. In this regard, arginine can be consumed by the enzymes iNOS, often expressed in tumour cells^48,49^, and by arginase, expressed by tumour-associated fibroblasts and macrophages (TAMs)^50^. Arginine is important for T and NK cell responses and arginine depletion in the tumour microenvironment has been shown to inhibit anti-tumour T cells responses^8,51–54^. Additionally, tryptophan can be depleted by the enzyme Indoleamine 2,3-dioxygenase (IDO), which is often highly expressed in tumour cells or in tumour-associated cells such as tolerogenic DCs^50,55^. IDO-mediated inhibition of T and NK cells is due to a combination of tryptophan depletion and the production of the metabolite kynurenine, which impacts the function of NK and T cells, at least in part, through acting upon the aryl hydrocarbon receptor (AhR)^56^. Originally, IDO was described as an innate mechanism of host defence against infection^57^. The effects of IDO activity on the local distribution of tryptophan and kynurenine is implicated in growth inhibition of certain bacteria, parasites and viruses^58,59^. The activity of IDO at these sites of infection will, therefore, also have implications for immune cells including T cells and NK cells.

Similarly to tumours, pathogens also utilise mechanisms to deprive immune cells of arginine as part of their immune evasion strategies. For example, *Helicobacter pylori* bacteria express arginase to deplete the local microenvironment of arginine and in doing so prevent iNOS-expressing macrophages from producing anti-microbial nitric oxide (NO)^60^. Low levels of systemic arginine and reduced NO production are also a feature of severe malarial infection^61^.

Therefore, there are multiple mechanisms that can result in the depletion of glucose and various amino acids in pathological immune microenvironments, and this can result in altered immune function and response to tumours or infection.

It is clear that tumours and pathogens compete with immune cells for nutrients as part of their immune evasion strategies, but equally there can be competition for nutrients between different immune cells, which may also be a normal physiological mechanism for regulating immune responses. Certainly, there are immunological situations where immune cells with elevated metabolism and nutrient demands compete with each other for the available fuels, such as within inflammatory lymph nodes where there is a rapid increase in the number of activated immune cells, or within the germinal centres where there is a concentration of metabolically active B cells and T follicular helper cells. Perhaps the best example where competition for nutrients between immune cells can play a role in shaping immune responses comes from studying DC–T cell interactions. There is evidence that an antigen-presenting DC can become starved of nutrients, such as glucose, due to competitive nutrient uptake by neighbouring cells, in particular activating CD8 T cells^25^. Interestingly, glucose deprivation of DC can result in increased DC proinflammatory outputs, including the expression of interleukin-12 and costimulatory molecules, which leads to enhanced CD8 T cell responses^25^.

**Fig. 3:**
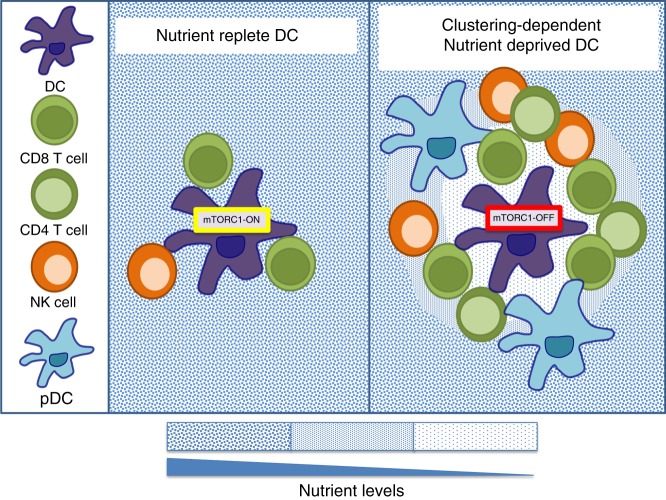
Competition for nutrients between immune cells. Antigen-presenting dendritic cells (DC) can be found at the centre of cell clusters consisting of numerous different types of activated immune cells, including CD8 T cells, CD4 T cells, NK cells and plasmacytoid dendritic cells (pDC), with elevated nutrient uptake rates that will compete for nutrients (blue dots). Depending on the number of clustering cells surrounding an antigen-presenting DC, nutrients may be available (left panel) or depleted (right panel) in the immediate surrounding microenvironment due to competitive uptake. Nutrient starvation will have consequences for the DC including the inactivation of mTORC1 signalling, which has been linked to increased proinflammatory DC functions

It is well established that T lymphocytes greatly increase nutrient uptake in response to antigen stimulation through up-regulating the expression of nutrient transporters. This is critically important in the generation of effector cells; indeed T cells lacking certain glucose or amino acid transporters fail to differentiate into effector cells. During activation, CD8 T cells cluster around antigen-presenting DCs within the lymph node^62–64^. These clustering T cells can potentially deplete the nutrients from the microenvironment surrounding the DCs (Fig. 3). In support of this, co-cultures of clustering CD8 T cells can inactivate the nutrient-sensitive mammalian Target of Rapamycin Complex 1 (mTORC1) signalling pathways in the interacting DCs^25^ (Fig. 3). In fact, antigen-presenting DCs can be found at the centre of cell clusters consisting of numerous different types of activated immune cells with elevated nutrient uptake rates in addition to CD8 T cells, including NK cells, CD4 T cells and pDC^65–68^. Therefore, it is tempting to speculate that starvation of DCs, and the resultant increase in DC outputs, is a physiological mechanism for the regulation of DC-induced T cells responses, a scenario where nutrients are acting as an immunological signal (Fig. 3). This is an interesting concept that remains to be formally tested.

Competition for nutrients between T cells has also been proposed as a mechanism for the selection of T cells that recognise antigen with high affinity^69^. Compared with those from low-affinity TCR, high-affinity TCR-antigen interactions induce a more robust and sustained metabolic response, with increased expression of glucose transporters and glycolytic genes^70^. Therefore, it is suggested that high-affinity T cell clones could outcompete their low-affinity counterparts for nutrients leading to nutrient starvation and apoptosis of these low-affinity T cell clones^69^. It is easy to imagine other situations where neighbouring immune cells would compete for nutrients in similar ways. For example, during B cell germinal centre responses, a solitary follicular helper T cell is surrounded by a large number of activating B cells with elevated nutrients demands. However, the inability to visualise nutrient abundance at the single-cell level represents a technical barrier that currently limits further exploration of nutrients as important immunological signals.

## Consequences of altered nutrient availability: signalling and immune outputs

**Fig. 4:**
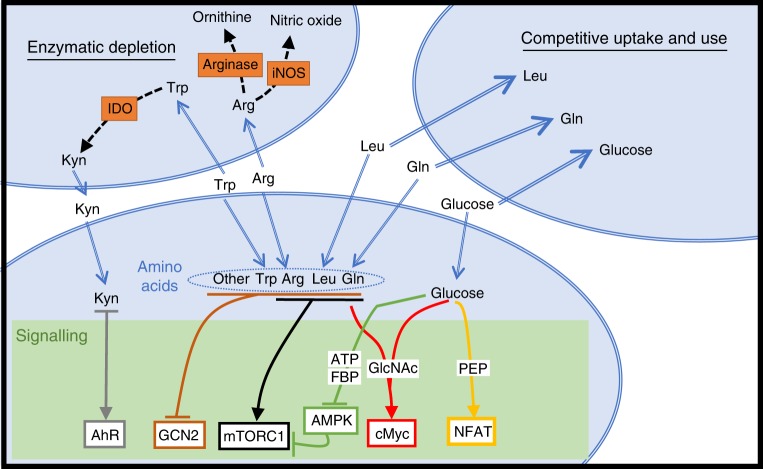
Competition for nutrients and the impact on signal transduction. Decreased levels of various nutrients within immune microenvironments could occur due to competitive uptake by surrounding cells. Alternatively, the expression of enzymes that consume nutrients, such as arginase, inducible nitric oxide synthase (iNOS) and Indoleamine-pyrrole 2,3-dioxygenase (IDO), can lead to reduced levels of arginine (Arg) and tryptophan (Trp). Limiting levels of nutrients will affect various signalling pathways. Mammalian target of rapamycin complex 1 (mTORC1) signalling is sensitive to levels of arginine, leucine (Leu) and glutamine (Gln). Glucose deprivation will also activate AMP-activated protein kinase (AMPK) due to reduced levels of ATP or fructose-1,6-bisphosphate (FBP) leading to the inhibition of mTORC1 activity. The metabolite phosphoenolpyruvate (PEP), generated when glucose is metabolised by glycolysis, can affect the duration of NFAT signalling. Gln and glucose are required for the production of uridine diphosphate *N*-acetylglucosamine (GlcNAc) that is important in sustaining the expression of the transcription factor cMyc. Decreased levels of amino acids in general will lead to the activation of general control nonderepressible 2 (GCN2). The product of IDO-mediated Trp metabolism, kynurenine (Kyn), can promote signalling through the aryl hydrocarbon receptor (AhR). NFAT nuclear factor of activated T cells

**Fig. 5:**
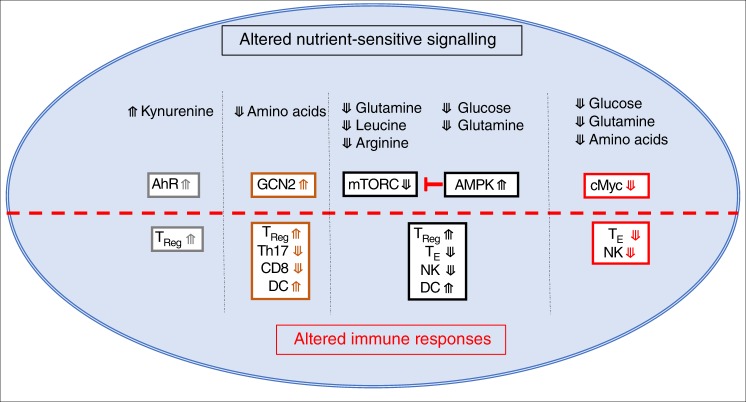
Immunological consequences of changes in nutrient signalling. Activation of AMP-activated protein kinase (AMPK) or inhibition of mammalian Target of Rapamycin Complex 1 (mTORC1) signalling promotes the differentiation of regulatory T (T_Reg_) cells over effector T cell subsets (T_E_), inhibits natural killer (NK) cell functions, and increases the proinflammatory outputs of dendritic cells (DC). Loss of cMyc expression inhibits the functions of T_E_ subsets and NK cells. Activation of general control nonderepressible 2 (GCN2) signalling promotes T_Reg_ differentiation, inhibits Th17 differentiation, inhibits CD8 T cell function, and enhances the function of DC. Kynurenine (Kyn)-mediated aryl hydrocarbon receptor (AhR) signalling promotes the differentiation of T_Reg_

Nutrient-restrictive microenvironments will directly impinge upon metabolic pathways in immune cells, but will also impact upon nutrient-sensitive signalling pathways important in immune regulation. Glucose and glutamine can impact multiple signalling pathways that are integral to the control of immune responses (Fig. 4). AMP-activated protein kinase (AMPK) is an indirect glucose sensor that becomes activated when ATP, or glycolytic intermediate fructose-1,6-bisphosphate, levels are decreased due to glucose restriction^71^. In effector T cells, AMPK can be activated within an hour of being placed in low concentrations of glucose^72,73^. Glutamine is also important for ATP production in effector T cells and AMPK can be activated by glutamine restriction in these cells^73^. AMPK negatively regulates the mTORC1, an important metabolic regulator with widespread roles in controlling immune cell functions^72–74^ (Fig. 4). Roles for mTORC1 include shaping T cell differentiation, controlling NK cells differentiation and effector function, and regulating the function of antigen-presenting DCs^74^ (Fig. 5). Therefore, the consequences of altered AMPK/mTORC1 signalling due to glucose restriction will include the inhibition inflammatory T cell and NK cell responses while promoting T_Reg_ differentiation (Fig. 5).

Interestingly, fructose-1,6-bisphosphate is not the only glycolytic intermediate that impacts important immune signalling pathways. Phosphenolpyruvate (PEP), another glucose metabolite, can affect Ca^2+^ signalling and the activation of the nuclear factor of activated T cells (NFAT) transcription factor in antigen-stimulated T cells (Fig. 4). PEP represses sarco/ER Ca^2+^-ATPase (SERCA) activity, which is responsible for Ca^2+^ reuptake into the ER; therefore, PEP prolongs cytosolic Ca^2+^ signalling and NFAT nuclear activity. T cells stimulated through the T cell antigen receptor in low glucose conditions, such as tumour microenvironments, have reduced PEP levels, reduced cytosolic Ca^2+^ signalling and less nuclear NFAT, leading to defective T cell activation^27^.

In addition to fueling glycolysis and OXPHOS, glucose and glutamine are also used for generation of uridine diphosphate *N*-acetylglucosamine (UDP-GlcNAc); this is the substrate for *O*-GlcNAcylation, which is the reversible addition of *N*-acetylglucosamine (GlcNAc) to proteins on serine or threonine residues by O-linked *N*-acetylglucosaminyltransferase (OGT). *O*-GlcNAcylation is dependent on the supply of both glucose and glutamine in T cells, suggesting that OGT and *O*-GlcNAcylation are important nutrient-sensing mechanisms in these cells^9^. Indeed, OGT is reported to be essential for normal T cell development, activation and clonal expansion^9,75^. Mechanistically, a number of signalling molecules that are important for T cell function are found to be *O*-GlcNAcylated, including c-Myc, NFAT and nuclear factor-κB^9,75,76^. This protein modification has not yet been studied in depth in other immune cell subsets.

Apart from glutamine, other amino acids also control numerous signalling pathways that are important for immune function. For example, the activity of mTORC1 is acutely sensitive to the levels of a number of amino acids including leucine, arginine and glutamine^77^. In addition, the transcription factor c-Myc is also regulated by amino acid availability. cMyc protein has a very short half-life in lymphocytes and sustained expression of cMyc is only possible in cells that have high rates of amino acid uptake and protein synthesis^6,9,19,78^. cMyc plays a crucial role during the activation and differentiation of T cell subsets and also of other lymphocytes including B cells and NK cells (Fig. 5)^11,19,78,79^. Moreover, the serine/threonine protein kinase General control nonderepressible 2 (GCN2) is a direct sensor of low cellular amino acid levels, and is activated through binding to uncharged transfer RNA (tRNA) (Fig. 4)^80^. GCN2 activity has been linked to the functions of various immune cells. In DCs, GCN2 activation results in enhanced antigen presentation to CD8 cells^81^. Conversely, GCN2 activity in gut antigen-presenting cells restrains excessive Th17 responses, with mice deficient of GCN2 developing stronger Th17 responses and more severe colitis in an induced colitis model^82^. IDO suppresses T cell responses, at least in part, by depleting tryptophan levels, leading to the activation of GCN2 within the T cell (Fig. 4). Activation of GCN2 in CD8 T cells results in proliferative arrest and anergy, while activation of GCN2 in CD4 T cells can lead to the generation of regulatory T cells (Fig. 5)^83,84^.

## The challenge of in vivo metabolic analysis

In vitro or ex vivo metabolic analyses have helped bring forth advances in our understanding of the metabolic phenotypes adopted by immune cells. While these studies have been extremely informative, the reported metabolic phenotypes may not be recapitulated in vivo. The metabolic phenotypes of immune cells are dependent on the supply of the relevant fuels such as glucose and glutamine, which are certainly less abundant in vivo than in culture conditions used in the laboratory. The consequence of a limiting supply of these fuels in vivo, within discrete immune microenvironments will be the restriction of metabolic pathways and the alteration of nutrient-sensitive signalling pathways that affect immune cell fate and function. However, our understanding of when and where nutrients are available in vivo is severely hampered by the lack of research tools to measure nutrient distribution at the single-cell level. Therefore, elucidating how nutrient supply affects the metabolism, signalling and thus function of immune cells in diverse and complex immune microenvironments remains a significant challenge for the immunometabolism field.
